# Physical frailty, genetic predisposition, and incident arrhythmias

**DOI:** 10.1002/jcsm.13499

**Published:** 2024-06-09

**Authors:** Yucong Zhang, Man Liu, Jiajun Li, Lei Ruan, Xiaofen Wu, Cuntai Zhang, Liangkai Chen

**Affiliations:** ^1^ Department of Geriatrics, Institute of Gerontology, Tongji Hospital of Tongji Medical College Huazhong University of Science and Technology Wuhan China; ^2^ Key Laboratory of Vascular Aging, Ministry of Education, Tongji Hospital of Tongji Medical College Huazhong University of Science and Technology Wuhan China; ^3^ Division of Cardiothoracic and Vascular Surgery, Tongji Hospital, Tongji Medical College Huazhong University of Science and Technology Wuhan China; ^4^ Department of Nutrition and Food Hygiene, Hubei Key Laboratory of Food Nutrition and Safety, School of Public Health, Tongji Medical College Huazhong University of Science and Technology Wuhan China; ^5^ Ministry of Education Key Lab of Environment and Health, School of Public Health, Tongji Medical College Huazhong University of Science and Technology Wuhan China

**Keywords:** Arrhythmias, Atrial fibrillation, Frailty, Prospective cohort, UK biobank

## Abstract

**Background:**

Cross‐sectional evidence suggests a possible link between frailty and atrial fibrillation (AF). It remains unclear whether frailty and incident arrhythmias are longitudinally associated. This study aimed to determine whether the frailty phenotype is longitudinally associated with incident arrhythmias, especially AF.

**Methods:**

In this prospective cohort of UK Biobank, individuals with arrhythmias at baseline, those without data for frailty phenotype, and no genetic data were excluded. Five domains of physical frailty, including weight loss, exhaustion, low physical activity, low grip strength, and slow gait speed, were assessed. A total of 142 single‐nucleotide polymorphisms was used to calculate the polygenic risk score (PRS) for AF. Hospital inpatient records and death records were used to identify incident arrhythmias.

**Results:**

This study included 464 154 middle‐aged and older adults (mean age 56.4 ± 8.1 years, 54.7% female) without arrhythmia at baseline. During a median follow‐up of 13.4 years (over 5.9 million person‐years), 46 454 new‐onset arrhythmias cases were recorded. In comparison with non‐frailty, the multivariable‐adjusted hazard ratios (HRs) of AF were 1.12 (95% CI: 1.09, 1.15, *P* < 0.0001) and 1.44 (95% CI: 1.36, 1.51, *P* < 0.0001) for participants with pre‐frailty and frailty, respectively. Similar associations were observed for other arrhythmias. We found that slow gait speed presented the strongest risk factor in predicting all arrhythmias, including AF (HR 1.34, 95% CI: 1.30, 1.39), bradyarrhythmias (HR 1.30, 95% CI: 1.22, 1.37), conduction system diseases (HR 1.29, 95% CI: 1.22, 1.36), supraventricular arrhythmias (HR 1.32, 95% CI: 1.19, 1.47), and ventricular arrhythmias (HR 1.37, 95% CI: 1.25, 1.51), with all *P* values <0.0001. In addition to slow gait speed, weight loss (HR 1.13, 95% CI: 1.09, 1.16, *P* < 0.0001) and exhaustion (HR 1.11, 95% CI: 1.07, 1.14, *P* < 0.0001) were significantly associated with incident AF, whereas insignificant associations were observed for physical activity (HR 1.03, 95% CI: 0.996, 1.08, *P* = 0.099) and low grip strength (HR 1.00, 95% CI: 0.97, 1.03, *P* = 0.89). We observed a significant interaction between genetic predisposition and frailty on incident AF (*P* for interaction <0.0001), where those with frailty and the highest tertile of PRS had the highest risk of AF (HR 3.34, 95% CI: 3.08, 3.61, *P* < 0.0001) compared with those with non‐frailty and the lowest tertile of PRS.

**Conclusions:**

Physical pre‐frailty and frailty were significantly and independently associated with incident arrhythmias. Although direct causal inference still needs to be further validated, these results suggested the importance of assessing and managing frailty for arrhythmia prevention.

## Introduction

Arrhythmias are highly prevalent in individuals of all ages, especially among older people.^1^ Among all types of arrhythmias, atrial fibrillation (AF) is the most typical sustained arrhythmia, affecting an estimated 2–4% of adults worldwide.^2^ The incidence of AF increases with age, rising from 1.5% in individuals aged 55–59 years to over 25% in those aged 85 years and older.^3^ Arrhythmias can lead to stroke, disability, and premature death, and intensifying trends of its prevalence will pose a tremendous economic and clinical burden.^1^ Therefore, early recognition and a better understanding of risk factors for arrhythmias is imperative.

Aging represents a principal risk factor for developing arrhythmia and frailty.^4^ Frailty is mainly characterized by the cumulative multisystem decline of physiological reserves that impairs an individual's ability to maintain homeostasis in response to stressors.^5^ Growing evidence suggests that frailty is associated with falls, functional disability, hospitalizations, and cardiovascular mortality and morbidity.^6^ Independent associations have been established between frailty and cardiovascular dysfunction, as well as abnormalities in cardiac structure and function, suggesting a potential role of frailty in arrhythmias.^7^ In AF patients, the pooled prevalence of frailty was reported to be 39.7%,^8^ with increasing frailty predicting heightened arrhythmia symptom severity and reduced quality of life.^4^ Increasing frailty was associated with a higher risk for all‐cause death and major adverse cardiovascular events in AF patients, with a non‐linear exponential relationship.^9^ Additionally, frailty is an independent predictor of all‐cause mortality and major bleeding, potentially related to anticoagulant treatment, in AF patients.^10^ However, limited longitudinal evidence exists regarding the association between frailty and subsequent risk of arrhythmias. As the commonest cardiac arrhythmia, the development of AF is affected by environment and genetic predisposition,^11^ although it remains unclear whether genetic susceptibility modifies the association between frailty and AF.

In this study, we investigated the association between baseline frailty and subsequent AF and other arrhythmias in a large prospective cohort. Moreover, we examined whether genetic risk modifies the association between frailty and incident AF.

## Methods

The statistical analysis plan was pre‐registered on Open Science Framework (Identifier: https://osf.io/4zq3c). Results were reported according to STrengthening the Reporting of OBservational studies in Epidemiology (STROBE) Checklists.

### Study population

More than 500 000 participants aged 40–69 years were included in this prospective cohort of UK Biobank. Individuals were enrolled from 2006 to 2010 at 22 assessment centres across England, Scotland, and Wales.^12^ In addition to touchscreen questionnaires, participants also received physical measurements. A baseline biological sample was collected from each participant. Informed consent was obtained from all individuals. This study was approved by the North West–Haydock Research Ethics Committee (21/NW/0157). Individuals with arrhythmias at baseline (*n* = 11 352), those without data for frailty phenotype (*n* = 16 696), and no genetic data (*n* = 10 196) were excluded. Finally, 464 154 participants were included in the current analysis (*Figure* *S1*).

### Assessment of frailty phenotype

Fried and colleagues^6^ described and applied the original frailty phenotype definition to the Cardiovascular Health Study, and the UK Biobank adapted the items^13^ [S1]. A detailed description of the frailty phenotype is presented in *Table* *S1*. Participants were classified as frailty (fulfilled three or more criteria), pre‐frailty (fulfilled one or two criteria), or non‐frailty (no criteria fulfilled).

### Ascertainment of outcomes

The primary outcome of interest was incident AF, and secondary outcomes included bradyarrhythmia, conduction system diseases, supraventricular arrhythmias, and ventricular arrhythmia. Arrhythmias were identified according to self‐reported medical history, hospital inpatient records, and national death registries. The detailed definitions of arrhythmias in the UK Biobank are provided in *Table* *S2*. A comparison was made between the dates of the first arrhythmia diagnosis and the entry into the study of participants to distinguish baseline or incident arrhythmia. At the time of analysis, data from hospital inpatient records were available until 31 October 2022 for England, 28 February 2018 for Wales, and 31 July 2021 for Scotland, that is, censored date.

### Covariates

At baseline, verbal interviews and touchscreen questionnaires were used to collect sociodemographic information, lifestyle factors, and health and medical history. Socioeconomic deprivation was assessed by the Townsend deprivation index.^14^ Participants were classified as ever, former, or current smokers for the assessment of smoking status. Participants were classified as never or special occasions only, one to three times a month, once or twice a week, three or four times a week, daily or most daily for the assessment of drinking patterns, which were calculated according to the frequency and alcohol equivalent of different drinks consumed on a typical day/week/month. The hours spent using computers, watching TV, and driving were summed up to calculate sedentary behaviour time.^15^ Participants' blood pressure, weight, and height were measured at their initial assessment centre visit by a trained nurse. Weight in kilograms was divided by the square of height in meters to calculate the BMI. Due to the potential effect of long‐term morbidities on frailty and adverse health outcomes^13^ [S2], the number of long‐term chronic diseases, such as cardiovascular and cerebrovascular diseases, heart diseases, metabolism disorders, respiratory diseases, and cancers, was assessed as a covariate, which was categorized as none, one, two, three, four, five and more. The detailed definitions of long‐term morbidities are shown in *Table* *S3*.

### Polygenic risk score for atrial fibrillation

The genotyping process, imputation, and quality control in the UK Biobank have been previously described.^16^ We applied 142 single nucleotide polymorphisms (SNPs) genome‐wide significantly associated with AF, which were identified in a genome‐wide association study comparing a total of 60 620 cases and 970 216 controls of European ancestry from six contributing studies.^17^ We calculated PRS using the following formula with each SNP weighted, that is, PRS = (β_1_ × SNP_1_ + β_2_ × SNP_2_ + … + β_n_ × SNP_n_) × (142/sum of the β coefficients), where β_n_ is the relative effect size, and SNP_n_ is the risk allele number of each SNP. As PRS increases, the genetic risk of developing AF increases, which is further divided into low, intermediate, and high tertiles. Detailed information about the selected SNPs is provided in *Table* *S4*, and the association between AF‐PRS and incident AF is presented in *Table* *S5*.

### Statistical analysis

A complete‐case analysis was performed. The association between frailty phenotype and arrhythmia subtypes was assessed using Cox proportional hazard regression models, and the proportional hazards assumption was met for all Cox models. The time to events was determined from the baseline recruitment date to the first‐time arrhythmia diagnosis or the date of loss to follow‐up, censoring, or death, whichever occurred first. In each model, frailty status was classified as frailty, pre‐frailty, or non‐frailty, in which non‐frailty was set as the referent, and hazard ratios (HRs) with 95% confidence intervals (CIs) were calculated. To examine whether the risk of arrhythmias increased linearly with the increasing number of frailty phenotype components, that is, the accumulative effect of frailty components, we treated the frailty phenotype score (from 0 to 5) as a continuous variable, and the *P* value for trend was calculated. We also assessed the association between five components of frailty and incident arrhythmia.

Genetic interaction analysis was performed on 440 365 participants of White British descent because AF‐PRS was derived from European ancestry. We set participants with non‐frailty and the lowest tertile of PRS as the referent to analyse the association between frailty and AF genetic risk with 3 × 3 groups. Using a fully adjusted model, including a multiplicative interaction term, frailty, and PRS tertiles were tested for their interaction effect. The association between frailty and AF stratified by tertiles of AF‐PRS was also assessed.

Several secondary analyses were performed. First, the associations between frailty and incident arrhythmia across strata of age, sex, Townsend deprivation index, and the number of long‐term morbidities were examined. Second, individuals who developed arrhythmias or died within 2 years from the baseline were excluded, and the main analyses were re‐run to minimize the potential reverse causation. Third, considering the competing mortality risk, the association was examined using the Fine&Grey subdistribution hazard model. Fourth, we further excluded patients with diagnosed cardiovascular disease, including coronary heart disease, heart failure, and stroke, and repeated the main analysis. Statistical analyses were conducted by SAS version 9.4 (SAS Institute Inc., Cary, NC, USA) and R software (the R Foundation, http://www.r‐project.org, version 4.2.0). A two‐sided *P* value <0.05 was considered statistically significant.

### Role of the funding source

The funders had no role in study design, data collection and analysis, decision to publish, or preparation of the manuscript.

## Results

### Baseline characteristics

Of the 464 154 included middle‐aged and older adults without arrhythmia at baseline (mean age 56.4 ± 8.1 years, 54.7% female), 273 498 (58.9%) were classified as non‐frailty, 174 589 (37.6%) were classified as pre‐frailty, and 16 067 (3.5%) were classified as frailty (*Table* *1*). Compared with non‐frailty, individuals with pre‐frailty or frailty were more likely to be female, non‐White, and current smokers. They also have higher BMI and longer sedentary behaviour but less alcohol consumption. In addition, they were more likely to have a larger number of long‐term morbidities, higher C‐reactive protein levels, and higher glycated haemoglobin levels at baseline.

**Table 1:** Baseline characteristics by frailty phenotype category.

	Frailty phenotype		
	Non‐frailty	Pre‐frailty	Frailty
Number of participants	273 498	174 589	16 067
Age, years	56.2 ± 8.1	56.6 ± 8.1	57.7 ± 7.6
Male	130 113 (47.6%)	74 396 (42.6%)	5893 (36.7%)
White	263 986 (96.5%)	162 221 (92.9%)	14 158 (88.1%)
Townsend deprivation index	−1.7 ± 2.9	−1.0 ± 3.2	0.6 ± 3.6
Smoking status			
Never	155 212 (56.8%)	92 570 (53.0%)	7419 (46.2%)
Former	93 940 (34.3%)	60 848 (34.9%)	5427 (33.8%)
Current	24 346 (8.9%)	21 171 (12.1%)	3221 (20.0%)
Alcohol consumption frequency			
Daily or most daily	62 541 (22.9%)	30 684 (17.6%)	1680 (10.5%)
Three or four times a week	71 649 (26.2%)	34 894 (20.0%)	1720 (10.7%)
Once or twice a week	71 718 (26.2%)	45 492 (26.1%)	3301 (20.5%)
One to three times a month	28 390 (10.4%)	21 550 (12.3%)	1984 (12.3%)
Never or special occasions only	39 200 (14.3%)	41 969 (24.0%)	7382 (45.9%)
Sedentary behaviour, hours/day	4.3 ± 2.4	4.8 ± 2.7	5.5 ± 3.4
BMI, kg/m^2^	26.6 ± 4.1	28.3 ± 5.1	31.2 ± 6.7
SBP, mm Hg	138.1 ± 18.6	137.6 ± 18.6	137.7 ± 18.9
DBP, mm Hg	82.3 ± 10.1	82.2 ± 10.2	82.2 ± 10.5
Glycated haemoglobin, %	5.4 ± 0.5	5.5 ± 0.7	5.8 ± 1.0
Triglycerides, mg/dL	149.9 ± 88.2	159.3 ± 93.0	178.5 ± 102.7
Total cholesterol, mg/dL	223.3 ± 42.9	217.8 ± 44.9	209.5 ± 48.8
High density lipoprotein cholesterol, mg/dL	57.4 ± 14.9	54.7 ± 14.4	51.1 ± 14.0
Low density lipoprotein cholesterol, mg/dL	139.5 ± 32.8	136.3 ± 34.1	130.7 ± 36.7
C‐reactive protein, mg/L	2.2 ± 3.7	2.9 ± 4.6	5.2 ± 7.3
The number of long‐term morbidities			
None	110 395 (40.4%)	48 186 (27.6%)	1423 (8.9%)
One	94 222 (34.5%)	56 921 (32.6%)	3119 (19.4%)
Two	46 094 (16.9%)	38 641 (22.1%)	3944 (24.5%)
Three	16 469 (6.0%)	19 152 (11.0%)	3414 (21.2%)
Four	4703 (1.7%)	7708 (4.4%)	2164 (13.5%)
Five and more	1615 (0.6%)	3981 (2.3%)	2003 (12.5%)

Values are mean ± SD or *n* (%), unless otherwise indicated.

BMI, body mass index; DBP, diastolic blood pressure; SBP, systolic blood pressure.

### Association between frailty phenotype and risk of arrhythmias

During a median follow‐up of 13.4 years (over 5.9 million person‐years), 46 454 new‐onset arrhythmias cases were documented, including 30 512 AF, 12 364 bradyarrhythmias, 12 901 conduction system diseases, 4134 supraventricular arrhythmias, and 4275 ventricular arrhythmias. In the fully adjusted model accounting for age, sex, race, Townsend deprivation index, assessment centres, alcohol consumption, smoking status, sedentary behaviour, BMI, the number of long‐term morbidities, AF‐PRS, the first 10 primary components of ancestry, and genotype measurement batches, in comparison with non‐frail individuals, the HRs of frailty were 1.44 (95% CI: 1.36, 1.51), 1.51 (95% CI: 1.39, 1.64), 1.56 (95% CI: 1.45, 1.69), 1.28 (95% CI: 1.10, 1.49), and 1.62 (95% CI: 1.42, 1.84) for AF, bradyarrhythmias, conduction system diseases, supraventricular arrhythmias, and ventricular arrhythmias, respectively (*Table* *2*). Similarly, the hazards of pre‐frail individuals were 1.12 (95% CI: 1.09, 1.15), 1.16 (95% CI: 1.12, 1.20), 1.20 (95% CI: 1.16, 1.25), 1.12 (95% CI: 1.05, 1.20), and 1.21 (95% CI: 1.13, 1.29) for AF, bradyarrhythmias, conduction system diseases, supraventricular arrhythmias, and ventricular arrhythmias, respectively (*Table* *2*).

**Table 2:** Association between frailty phenotype and incident arrhythmia.

	Non‐frailty	Pre‐frailty	Frailty
AF			
Incident rate per 10 000 py	44.2	5.83	103.1
Model 1	1.00 (ref.)	1.32 (1.29, 1.35)	2.33 (2.23, 2.45)
Model 2	1.00 (ref.)	1.12 (1.09, 1.15)	1.44 (1.36, 1.51)
Bradyarrhythmias			
Incident rate per 10 000 py	17.5	23.5	40.8
Model 1	1.00 (ref.)	1.36 (1.31, 1.41)	2.40 (2.22, 2.59)
Model 2	1.00 (ref.)	1.16 (1.12, 1.20)	1.51 (1.39, 1.64)
Conduction system diseases			
Incident rate per 10 000 py	17.4	25.4	47.6
Model 1	1.00 (ref.)	1.45 (1.40, 1.50)	2.70 (2.51, 2.90)
Model 2	1.00 (ref.)	1.20 (1.16, 1.25)	1.56 (1.45, 1.69)
Supraventricular arrhythmias			
Incident rate per 10 000 py	6.1	7.6	11.3
Model 1	1.00 (ref.)	1.23 (1.15, 1.31)	1.76 (1.53, 2.03)
Model 2	1.00 (ref.)	1.12 (1.05, 1.20)	1.28 (1.10, 1.49)
Ventricular arrhythmias			
Incident rate per 10 000 py	5.8	8.3	16.3
Model 1	1.00 (ref.)	1.43 (1.34, 1.52)	2.72 (2.41, 3.07)
Model 2	1.00 (ref.)	1.21 (1.13, 1.29)	1.62 (1.42, 1.84)

Model 1, included age, sex, race, Townsend deprivation index, and assessment centres. Model 2, included Model 1 plus alcohol consumption, smoking status, sedentary behaviour, BMI, the number of long‐term morbidities, AF‐PRS, the first 10 primary components of ancestry, and genotype measurement batches.

AF, atrial fibrillation/flutter; PRS, polygenic risk score; py, person‐years.

**Figure 1:**
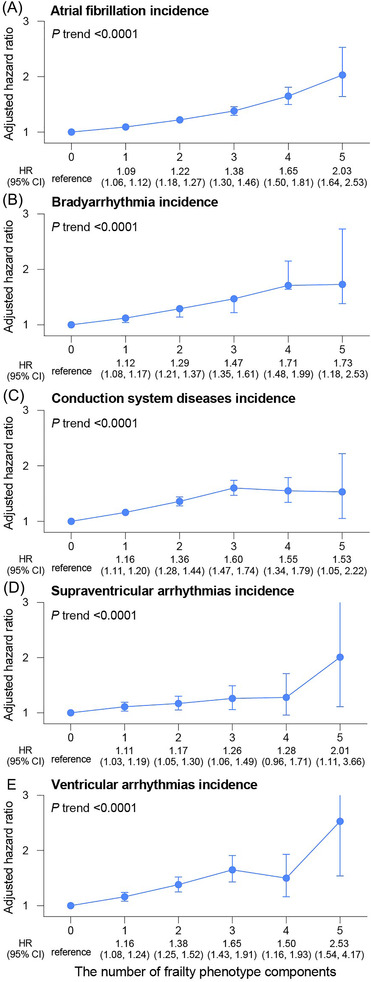
Associations between the cumulative number of frailty components and the incidence of atrial fibrillation (A), bradyarrhythmia (B), conduction system diseases (C), supraventricular arrhythmias (D), and ventricular arrhythmia (E).

Positive linear associations between the cumulative number of frailty components and the incidence of AF and other subtypes of arrhythmias were observed (all linear trend tests *P* < 0.0001, *Figure* *1*). With each additional increase in the component of the frailty, the HRs increased by 12% (95% CI: 1.10, 1.13), 14% (95% CI: 1.11, 1.16), 15% (95% CI: 1.13, 1.17), 8% (95% CI: 1.05, 1.13), and 16% (95% CI: 1.12, 1.20) for AF, bradyarrhythmias, conduction system diseases, supraventricular arrhythmias, and ventricular arrhythmias, respectively.

### Association between frailty components and risk of arrhythmias

We investigated the association between the five components of frailty and incident arrhythmias independently and mutually (*Table* *S6*). In the fully adjusted model without mutual adjustment for these five frailty components (Model 2), we found that weight loss (HR 1.12, 95% CI: 1.09, 1.16), exhaustion (HR 1.15, 95% CI: 1.11, 1.19), low physical activity (HR 1.12, 95% CI: 1.07, 1.16), low grip strength (HR 1.04, 95% CI: 1.01, 1.07), and slow gait speed (HR 1.37, 95% CI: 1.33, 1.42) were significantly associated with AF. Similar associations were found between these frailty components and other subtypes of arrhythmias (*Table* *S6*). In the mutual adjustment model (Model 3 in *Table* *S6*), we found that slow gait speed remained the strongest risk factor in predicting all arrhythmias, including AF (HR 1.34, 95% CI: 1.30, 1.39), bradyarrhythmias (HR 1.30, 95% CI: 1.22, 1.37), conduction system diseases (HR 1.29, 95% CI: 1.22, 1.36), supraventricular arrhythmias (HR 1.32, 95% CI: 1.19, 1.47), and ventricular arrhythmias (HR 1.37, 95% CI: 1.25, 1.51).

### Interaction between frailty and polygenic risk score for atrial fibrillation on incident atrial fibrillation

**Figure 2:**
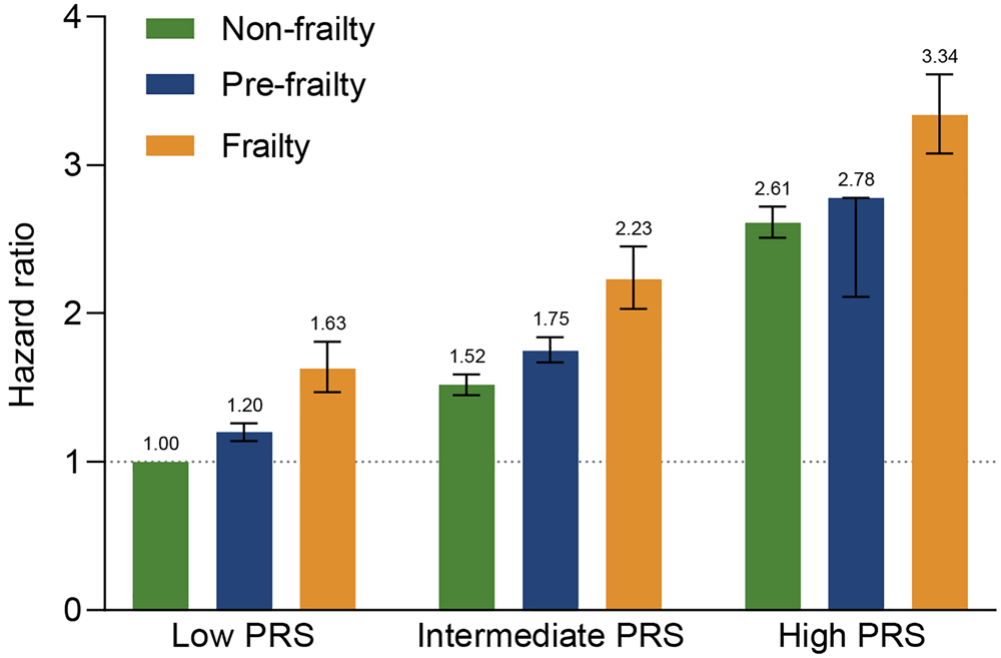
Risk of incident atrial fibrillation according to genetic risk and frailty phenotype. PRS = polygenic‐risk score.

Compared with the low PRS group, the HRs of individuals with intermediate PRS and high PRS were 1.48 (95% CI: 1.43, 1.52) and 2.43 (95% CI: 2.36, 2.50) for AF, respectively (*Table* *S5*). Compared with non‐frailty, people with frailty had an increased risk of incident AF by 36% (HR 1.36, 95% CI: 1.26, 1.48), 40% (HR 1.40, 95% CI: 1.27, 1.54), and 53% (HR 1.53, 95% CI: 1.37, 1.71) in low, intermediate, and high PRS groups, respectively, and we observed a significant interaction (*P* < 0.0001) in such association (*Table* *S7*). We further assessed the joint association between frailty phenotype and PRS on incident AF (*Figure* *2*). Participants with frailty and the highest tertile of PRS had the highest risk of AF (adjusted HR 3.34, 95% CI: 3.08, 3.61) compared with those with non‐frailty and the lowest tertile of PRS.

### Secondary analyses

The associations of frailty and incident arrhythmias remained persistent in most subgroups in the stratus across age (<55, 55–64, and ≥65 years), sex (male and female), Townsend deprivation index (dichotomous), BMI (normal weight, overweight, and obesity), and the number of long‐term morbidities (none, one, two, or more) (*Tables* *S8–S12*). To minimize the potential reverse causality, individuals who developed arrhythmias or died within 2 years of follow‐up were excluded, and the associations of frailty and arrhythmias incidence were not materially altered (*Table* *S13*). Such associations remained robust in the Fine&Grey competing models (*Table* *S14*), and in participants free of diagnosed cardiovascular disease (*Table* *S15*).

## Discussion

In this study of 464 154 middle‐aged and older adults in the UK Biobank without arrhythmias at baseline, the prevalence of pre‐frailty and frailty was 37.6% and 3.5%, respectively. In comparison with non‐frailty, individuals with pre‐frailty had a 12% to 21% and frailty had a 28% to 62% increased risk of arrhythmias, including AF, bradyarrhythmia, conduction system diseases, supraventricular arrhythmias, and ventricular arrhythmia. These associations were independent of socioeconomic factors, lifestyles, morbidities, and genetic risk of AF.

The role of frailty in AF patients has been investigated. A systematic review demonstrated that frailty is found in 4.4–75.4% of patients with AF, while AF affects 48.2–75.4% of patients with frailty.^7^ Frailty is reported to independently predict higher arrhythmia symptom intensity and worse quality of life in older patients with AF.^4^ In this study, frailty was assessed by using the Edmonton Frail Scale, which contains 10 domains associated with balance, health attitudes, cognitive function, nutrition, social support, mobility, mood, quality of life, functional independence, and medication. Moreover, a study enrolled 10 177 AF patients, of whom 6066 (59.6%) were pre‐frail and 2172 (21.3%) were frail, which were assessed by a 40‐item frailty index.^9^ Furthermore, frailty is even reported in association with an elevated risk of ischemic stroke, bleeding,^8^ and even all‐cause death for AF patients.^8^, ^10^ Unfortunately, the longitudinal association between frailty and the risk of incident AF and other subtypes of arrhythmias remains controversial or unknown. A longitudinal study from the Kuopio Ischemic Heart Diseases Risk Factor Study involving 839 participants suggested that frailty could predict the development of AF in women but not in men [S3]. Conversely, findings from another longitudinal study based on the Framingham Heart Study offspring cohort, which included approximately 2000 participants for analysis, failed to establish a statistically significant association between frailty and incident AF [S4]. Interestingly, despite the lack of statistical significance, both studies hinted at a trend wherein frail individuals faced an elevated risk of AF onset, potentially constrained by limited sample sizes and new‐onset cases. In our study, frailty was defined by Fried based on physical phenotypes.^6^ It is relatively easy and affordable to conduct and establish a basis for standardized frailty screening due to its clear criteria. It has also been considered one of the most commonly used frailty assessment instruments^18^ [S5]. We first reported the longitudinal association between pre‐frailty and frailty and subsequent AF risk. Additionally, such association remained robust after considering a wealth of covariates, including age, sex, race, socioeconomic status (i.e., Townsend deprivation index), lifestyles (i.e., alcohol consumption, smoking status, sedentary behaviour), BMI, multiple long‐term morbidities, and genetic predisposition. Moreover, the associations of frailty and incident AF and other arrhythmias remained robust across diverse subgroups stratified by age, sex, Townsend deprivation index, BMI, and the number of long‐term morbidities.

Consistent findings regarding the relationship between frailty and other arrhythmia subtypes have emerged, including bradyarrhythmias, conduction system diseases, supraventricular arrhythmias, and ventricular arrhythmias, which were previously overlooked in studies solely focused on frailty and AF^7^ [S6]. A study assessing frailty using the short physical performance battery found that the majority (71%) of older patients requiring pacemaker implantation due to bradyarrhythmia were frail, and frailty was associated with an elevated risk of mortality.^19^ However, there is a paucity of research exploring the association between frailty and the incidence of bradyarrhythmia. Similarly, while it was observed that 33.4% of patients with atrioventricular block who qualified for pacemaker implantation were frail, as assessed by the Canadian Study of Health and Aging Clinical Frailty Scale, the link between frailty and the development of conduction system impairment remains unexplored.^20^ Furthermore, despite the higher prevalence of premature ventricular complexes, which are indicative of life‐threatening arrhythmias such as sustained ventricular tachycardia and ventricular fibrillation, in older individuals,^21^ and the notable frailty among most ventricular arrhythmia patients referred for consideration of implantable cardioverter‐defibrillator,^22^ investigations into the associations between frailty and ventricular arrhythmias have been lacking. Hence, future research should delve into the roles of frailty in the development and outcomes of arrhythmias beyond AF.

In this study, weight loss, exhaustion, low grip strength, and slow gait speed were independently associated with an elevated risk of incident arrhythmia. Being underweight is reported to be a risk factor for incident AF and poorer cardiovascular outcomes^23^ [S7]. One study demonstrated that a one‐unit decrease in BMI was associated with a 13% elevation of AF risk.^24^ Besides, being underweight was also related to an increased AF recurrence rate after catheter ablation.^25^ Lower gait speed is common in patients with cardiac diseases. In particular, AF is significantly associated with lower usual gait speed, especially for those aged 70 or older. Therefore, AF may be associated with an elevated risk of falls, hospitalization, cognitive decline, stroke, heart failure, and even mortality.^26^ Although lower gait speed is reported to increase the risk of major morbidities after cardiac operations, such as prolonged intubation, deep sternal wound infection, postoperative renal failure, surgical reexploration, and stroke,^27^ unfortunately, there is a lack of study that analysed the role of low gait speed in the risk of incident AF. In our study, lower gait speed is the strongest predictor for all types of arrhythmias among all frailty components. Higher muscle strength was reported to be related to a decreased risk of bradyarrhythmia and ventricular arrhythmia or cardiac arrest.^26^ However, no associations between muscle strength and AF or supraventricular arrhythmia were found.^26^ Overall, studies on the associations between specific frailty components and specific arrhythmia subtypes are limited. Our results filled this gap and offered a comprehensive picture of the frailty phenotype and its components as the risk factor of arrhythmia.

In this study, a panel of 142 SNPs was employed to construct AF‐related PRS and elucidate the modifying effect of genetic predisposition on frailty and incident AF. Our findings demonstrated an elevated risk of incident AF associated with a higher AF‐PRS and a more pronounced frail status. Moreover, significant interactions between frailty and PRS categories were observed in both combined and stratified analyses, highlighting a complex interplay between genetic and environmental factors in the occurrence of AF. Notably, individuals classified as non‐frailty exhibited a significantly diminished risk of incident AF across all PRS tertiles. These results underscore the potential advantages of enhancing physical frailty, particularly among individuals with an augmented genetic susceptibility to AF.

Although frailty has not been routinely assessed in clinical practice, a growing number of clinical guidelines recognize the importance of frailty assessment^28^ [S8]. Our study encourages the integration of frailty management into arrhythmia prevention. In addition to frailty, we found that more than a third of individuals (37.6%) were in a pre‐frailty state. Pre‐frailty is considered potentially reversible as a highly prevalent intermediate state before frailty. Individuals with pre‐frailty have 2.63 times the risk for developing frailty compared with those without frailty at baseline.^6^ Our study also found a significant association between pre‐frailty and elevated risk of arrhythmia, highlighting the significance of managing pre‐frailty. Strategies based on exercise‐based, educational, and nutritional interventions in primary care may help delay or even reverse frailty^29^ [S9]. With the development of society and the progress of life and medical science, health expectancy has attracted more attention than life expectancy^30^ [S10, S11]. Arrhythmia and its co‐morbidities are well‐recognized threats to health, leading to incapacity or even fatal diseases. According to these results, frailty assessment and management should be included in the personalizing assessment for middle and older individuals. Furthermore, akin to frailty, intrinsic capacity is a novel concept recently introduced by the World Health Organization. It encompasses elements from the activity, body function, and structure domains, aiming to evaluate the remaining biological capabilities of older individuals.^31^ Although impairments in domains that are potentially associated with intrinsic capacity have been frequently considered as constituents of frailty, it is important to note that while frailty is characterized by reduced strength, endurance, and physiological function, intrinsic capacity represents the collective physical and mental capacities of an individual. Consequently, employing a comprehensive assessment that combines both frailty and intrinsic capacity may better cater to and address the specific needs and priorities of individuals.^32^

Strong associations exist between frailty and multi‐morbidity, both of which contribute to the occurrence of arrhythmias. In our study, the robust association between frailty and arrhythmia incidence persisted even after adjusting for the number of long‐term conditions, suggesting the presence of additional underlying mechanisms linking frailty to arrhythmia risk beyond the existing co‐morbidities. Findings from the Cardiovascular Health Study indicate that inflammation and coagulopathy play pivotal roles in the physiological basis of frailty, independent of co‐morbidities.^33^ The inflammatory response and immune system pathways are implicated in various cardiovascular pathophysiological conditions, including cardiac arrhythmias.^34^ The inflammation process involves a variety of cytokines produced via distinct molecular pathways, exerting proarrhythmic effects. Additionally, excessive reactive oxygen species generated primarily by mitochondria disrupt cardiac myocyte energy production, redox balance, and cellular electrophysiology, contributing to the development of cardiac arrhythmias.^35^ Frailty is characterized by a homeostatic imbalance at the molecular and physiological levels, with oxidative stress resulting from mitochondrial dysfunction impairing various physiological processes and triggering the release of inflammatory mediators directly associated with frailty.^36^ The combination effects of inflammation and oxidative stress might serve as pathophysiological mechanisms for frailty. Assessing the levels of inflammation and oxidative stress in future studies would be valuable for establishing the underlying mechanism of frailty and arrhythmias. Notably, proleptic aging, characterized by chronic inflammation and mitochondrial dysfunction, presents a common physiological basis for cardiac arrhythmias and frailty.^37^ Therefore, the relationship between arrhythmia and frailty might be bidirectional, influenced by the cumulative burden of shared risk factors.^38^

Our study has several strengths. Considering the limited longitudinal evidence on the association between physical frailty and incident arrhythmias, we provided the first‐large prospective evidence in this regard. The long‐term follow‐up and large sample size strengthen the credibility of our results. Furthermore, the UK Biobank provides overwhelming information that allows us to assess many confounders, especially the genetic predisposition of AF.

### Study limitations

First, due to the unavailability of data in the UK Biobank, certain risk factors associated with acquired arrhythmias, such as a history of electrolyte disturbance and toxic exposure, could not be considered. Second, the UK Biobank had a low response rate (5.47%) and selection bias. Nevertheless, recent studies have indicated that risk factor associations seem generalizable despite a meagre response rate in the UK Biobank.^39^ Third, we excluded many participants due to missing data on frailty assessments and covariates, which may bring potential bias. A complete‐case analysis was conducted, which provided biased estimates when the data were not missing completely randomly. However, all data related to the variables involved in our analysis were not missing. Fourth, reporting bias may exist due to some self‐reported covariates and frailty components, which might complicate result interpretation. However, a study has indicated that regardless of whether test‐based or self‐reported measures are employed, the characteristics of frailty remain largely consistent.^40^ Fifth, only Whites were included in our study, and the associations between frailty and arrhythmias should be warranted and replicated in other ethnicities. Sixth, given the challenge of conducting repeated measurements in an extremely large cohort study, most research studies, including the UK Biobank, rely on baseline frailty status to predict future disease risks, which precludes assessing the association between transitions in frailty and the risk of arrhythmias. Seventh, while the assessment of physical activity through self‐reporting may introduce certain biases and restrict the evaluation of behavioural changes, we employed the validated International Physical Activity Questionnaire, which is able to reflect an individual's exercise habits. Eighth, the determination of arrhythmia incidence involved the application of a series of algorithms, and diagnosis bias may not be completely ruled out, which may raise concerns regarding potential diagnostic bias and missed diagnoses. However, a prior publication has demonstrated the relative reliability of our chosen methodology [S12]. Finally, our observation of a higher prevalence of obesity and co‐morbidities among frail individuals raises considerations for cautious interpretation. From a causal inference perspective, frailty can be considered as a complex pathophysiological consequence arising from confounding pathological conditions, rather than necessarily a cause. Nevertheless, our subgroup analyses revealed a robust association of pre‐frailty and frailty with AF and other arrhythmias, irrespective of individual obesity status or the presence of multiple co‐morbidities. Nonetheless, it is imperative to exercise caution when inferring direct causal relationships due to the observational nature of our study.

## Conclusions

In this large study over 13 years of follow‐up, pre‐frailty and frailty were significantly and independently associated with the incidence of arrhythmias. Moreover, individuals with frailty and a high genetic risk of AF have the highest risk of AF. Considering frailty management as a part of the primary prevention of arrhythmias may help identify high‐risk individuals. Future studies are needed to investigate the direct causal inference, and the role of frailty management in the prevention of arrhythmias.

## Funding

C.Z. is supported by the National Key Research and Development Program of China (grant number 2020YFC2008000) and the Key Research and Development Program of Hubei Province (grant number 2022BCA001). L.C. is supported by the Young Elite Scientists Sponsorship Program by China Association for Science and Technology (grant number YESS20210143).

## Conflict of interest

The authors declared no conflict of interests.

## Supporting information


**Table S1.** Frailty Definition and Cut‐off Points in UK Biobank Study
**Table S2.** Definitions of Arrhythmia in UK Biobank Study
**Table S3.** Definition and List of Long‐Term Morbidities
**Table S4.** Information of 142 SNPs for AF‐PRS in UK Biobank
**Table S5.** Association between AF‐PRS and Incident AF (*n* = 440,365)
**Table S6.** Individual Components of Frailty Phenotype and Their Association with Incident Arrhythmia
**Table S7.** Risk of Incident AF According to Frailty Status within the AF‐PRS Category (*n* = 440,365)
**Table S8.** Association between Frailty and Incident AF by Subgroups
**Table S9.** Association between Frailty and Incident Bradyarrhythmia by Subgroups
**Table S10.** Association between Frailty and Incident Conduction System Diseases by Subgroups
**Table S11.** Association between Frailty and Incident Supraventricular Arrhythmias by Subgroups
**Table S12.** Association between Frailty and Incident Ventricular Arrhythmia by Subgroups
**Table S13.** Association between Frailty and Incident Arrhythmias Excluding Those Who Developed Arrhythmias or Died within Two Years of Follow‐up (*n* = 458,781)
**Table S14.** Association between Frailty and Incident Arrhythmias Using Fine & Grey Models for Competing Risk
**Figure S1.** Flow chart
**Figure S2.** The Curve of Density Distribution of AF‐PRS
