# Supplementary material for: Physical frailty, genetic predisposition, and incident arrhythmias
Source: J Cachexia Sarcopenia Muscle. 2024 Jun 9;15(4):1463–72. doi: 10.1002/jcsm.13499 (PMC11294030; doi:10.1002/jcsm.13499)
Supplement: Supplementary file 1 — Table S1. Frailty Definition and Cut‐off Points in UK Biobank Study Table S2. Definitions of Arrhythmia in UK Biobank Study Table S3. Definition and List of Long‐Term Morbidities Table S4. Information of 142 SNPs for AF‐PRS in UK Biobank Table S5. Association between AF‐PRS and Incident AF (n = 440,365) Table S6. Individual Components of Frailty Phenotype and Their Association with Incident Arrhythmia Table S7. Risk of Incident AF According to Frailty Status within the AF‐PRS Category (n = 440,365) Table S8. Association between Frailty and Incident AF by Subgroups Table S9. Association between Frailty and Incident Bradyarrhythmia by Subgroups Table S10. Association between Frailty and Incident Conduction System Diseases by Subgroups Table S11. Association between Frailty and Incident Supraventricular Arrhythmias by Subgroups Table S12. Association between Frailty and Incident Ventricular Arrhythmia by Subgroups Table S13. Association between Frailty and Incident Arrhythmias Excluding Those Who Developed Arrhythmias or Died within Two Years of Follow‐up (n = 458,781) Table S14. Association between Frailty and Incident Arrhythmias Using Fine & Grey Models for Competing Risk Figure S1. Flow chart Figure S2. The Curve of Density Distribution of AF‐PRS [file JCSM-15-1463-s001.docx]

**Physical Frailty, Genetic Predisposition, and Incident Arrhythmias**

**Table S1. Frailty Definition and Cut-off Points in UK Biobank Study**

| **Individual components** | **Criteria** | **Field IDs** |
| --- | --- | --- |
| **Weight loss** | Self-reported: “Compared with one year ago, has your weight changed?”  Response:   - Yes, lost weight = 1; - Other = 0; - Prefer not to answer = missing data. | 2306 |
| **Exhaustion** | Self-reported: “Over the past two weeks, how often have you felt tired or had little energy?”  Response:   - More than half the days or nearly every day = 1; - Other = 0; - Prefer not to answer = missing data. | 2080 |
| **Low physical activity** | Self-reported: “In the last 4 weeks did you spend any time doing the following? (You can select more than one answer)”  Response:   - Walking for pleasure (not as a means of transport) = 0; - Other exercises (eg: swimming, cycling, keep fit, bowling) = 0; - Strenuous sports = 0; - Light DIY (eg: pruning, watering the lawn): - Frequency of once per week or less=1; - Frequency of more than once per week=0; - Heavy DIY (eg: weeding, lawn mowing, carpentry, digging) = 0; - None of the above = 1; - Prefer not to answer = missing data. | 6164, 1011 |
| **Slow gait speed** | Self-reported: “How would you describe your usual walking pace?”  Response:   - Slow = 1; - Other = 0; - Prefer not to answer = missing data. | 924 |
| **Low grip strength** | Measured grip strength expressed in kg by sex- and BMI- adjusted cut-off points.  Cut-off points:  Men   - If BMI ≤24.0 kg/m^2^ & grip strength ≤29 kg - If BMI 24.1 to 28.0 kg/m^2^ & grip strength ≤30 kg - If BMI >28.0 kg/m^2^ & grip strength ≤32 kg   Women   - If BMI ≤23.0 kg/m^2^ & grip strength ≤17 kg - If BMI 23.1 to 26.0 kg/m^2^ & grip strength ≤17.3 kg - If BMI 26.1 to 29.0 kg/m^2^ & grip strength ≤18 kg - If BMI >29.0 kg/m^2^ & grip strength ≤21 kg - If data on BMI or grip strength is not available = missing data. | 31, 21001, 46, 47 |

Abbreviations: BMI, body mass index; MET, metabolic equivalent of energy; IPAQ, International Physical Activity Questionnaire.

**Table S2. Definitions of Arrhythmia in UK Biobank Study**

|  | **ICD-9** | **ICD-10** | **OPCS-4** | **Self-reported fields** |
| --- | --- | --- | --- | --- |
| **Atrial Fibrillation/Flutter** | 4273 | I48, I48.0, I48.1, I48.2, I48.3, I48.4, I48.9 | K62.1, K62.2, K62.3 | 20002 (1471, 1483) |
| **Ventricular Arrhythmia** | 4271, 4274 | I47.0, I47.2, I49.0, I46.0, I46.1, I46.9 | K57.6, K64.1, X50.3, X50.4, X50.8, X50.9 |  |
| **Bradyarrhythmia** | 4260, 4261, 4266 | I44.0, I44.1, I44.2, I44.3, I44.5, I49.5 | K60, K60.1, K60.2, K60.3, K60.4, K60.5, K60.6, K60.8, K60.9, K61, K61.1, K61.2, K61.3, K61.4, K61.5, K61.6, K61.8, K61.9 | 20002 (1486) |
| **Conduction system diseases** | 4263, 4264, 4265 | I44.0, I44.4, I44.5, I44.6, I44.7, I45.0, I45.1, I45.2, I45.3, I45.4 |  |  |
| **Supraventricular arrhythmias** | 4267, 4270 | I45.6, I47.1, I49.1 | K52.4, K57.2, K57.4, K57.5 | 20002 (1484, 1487) |

Variable definitions constructed using ICD-9, ICD-10 codes, and self-reported data fields with the choice-, disease- or procedure-specific codes between brackets are shown.

Abbreviations: ICD, International Classification of Diseases.

**Table S3. Definition and List of Long-Term Morbidities**

|  | **Morbidity grouping*** | **Conditions included** | **Code** |
| --- | --- | --- | --- |
| **1** | Hypertension | Hypertension | 1065 |
|  |  | Essential hypertension | 1072 |
| **2** | Coronary heart disease | Heart attack/MI | 1075 |
|  |  | Angina | 1074 |
| **3** | Diabetes | Diabetic nephropathy | 1607 |
|  |  | Diabetic neuropathy/ulcers | 1468 |
|  |  | Diabetes | 1220 |
|  |  | Type 1 diabetes | 1222 |
|  |  | Type 2 diabetes | 1223 |
|  |  | Diabetic eye disease | 1276 |
| **4** | Stroke/TIA | Stroke | 1081 |
|  |  | TIA | 1082 |
|  |  | Subarachnoid haemorrhage | 1086 |
|  |  | Brain haemorrhage | 1491 |
|  |  | Ischaemic stroke | 1583 |
| **5** | Atrial fibrillation | Atrial fibrillation | 1471 |
| **6** | Heart failure | Cardiomyopathy | 1079 |
|  |  | Hypertrophic cardiomyopathy | 1588 |
|  |  | Heart failure/pulmonary oedema | 1076 |
| **7** | Peripheral vascular disease | Peripheral vascular disease | 1067 |
|  |  | Leg claudication/intermittent claudication | 1087 |
| **8** | COPD | COPD/Chronic obstructive pulmonary disease | 1112 |
|  |  | Emphysema/Chronic bronchitis | 1113 |
|  |  | Emphysema | 1472 |
| **9** | Asthma | Asthma | 1111 |
| **10** | Bronchiectasis | Bronchiectasis | 1114 |
| **11** | Cancer* | “yes”/”no” to “have you ever had cancer?” |  |
| **12** | Dyspepsia | Gastro-oesophageal reflux (GORD) | 1138 |
|  |  | Oesophagitis/Barrett’s oesophagus | 1139 |
|  |  | Gastric stomach ulcers | 1142 |
|  |  | Gastric erosions/gastritis | 1143 |
|  |  | Duodenal ulcer | 1457 |
|  |  | Dyspepsia/indigestion | 1510 |
|  |  | Hiatus hernia | 1474 |
|  |  | Helicobacter pylori | 1442 |
| **13** | Diverticular disease | Diverticular disease/diverticulitis | 1458 |
| **14** | Irritable bowel syndrome | Irritable bowel syndrome | 1154 |
| **15** | Chronic liver disease | Oesophageal varices | 1141 |
|  |  | Non infective hepatitis | 1157 |
|  |  | Liver failure/cirrhosis | 1158 |
|  |  | Primary biliary cirrhosis | 1506 |
| **16** | Inflammatory bowel disease | Inflammatory bowel disease | 1461 |
|  |  | Crohn’s disease | 1462 |
|  |  | Ulcerative colitis | 1463 |
| **17** | Constipation | Constipation | 1599 |
| **18** | Viral hepatitis | Hepatitis B | 1579 |
|  |  | Hepatitis C | 1580 |
|  |  | Hepatitis D | 1581 |
| **19** | Depression | Depression | 1286 |
|  |  | Postnatal depression | 1531 |
| **20** | Anxiety | Anxiety/panic attacks | 1287 |
|  |  | Nervous breakdown | 1288 |
|  |  | Post-traumatic stress disorder | 1469 |
|  |  | Obsessive compulsive disorder | 1615 |
|  |  | Stress | 1614 |
|  |  | Insomnia | 1616 |
|  |  | Psychological/psychiatric problem | 1243 |
| **21** | Schizophrenia/Bipolar affective disorder | Scizophrenia | 1289 |
|  | Bipolar | Mania | 1291 |
|  |  | Bipolar disorder | 1291 |
|  |  | Manic depression | 1291 |
| **22** | Connective tissue diseases | Myositis/myopathy | 1322 |
|  |  | Systemic lupus erythematosus/SLE | 1381 |
|  |  | Connective tissue disorder | 1373 |
|  |  | Sjogren’s syndrome.sicca syndrome | 1382 |
|  |  | Dermatopolymyositis | 1383 |
|  |  | Scloeroderma/systemic sclerosis | 1384 |
|  |  | Rheumatoid arthritis | 1464 |
|  |  | Psoriatic arthropathy | 1477 |
|  |  | Dermatomyositis | 1480 |
|  |  | Polymyositis | 1481 |
|  |  | Polymyalgia rheumatica | 1377 |
| **23** | Painful conditions | Back pain | 1534 |
|  |  | Joint pain | 1537 |
|  |  | Headaches (not migraine) | 1436 |
|  |  | Sciatica | 1476 |
|  |  | Plantar fasciitis | 1540 |
|  |  | Carpal tunnel syndrome | 1541 |
|  |  | Fibromyalgia | 1542 |
|  |  | Arthritis | 1538 |
|  |  | Shingles | 1573 |
|  |  | Disc problem | 1532 |
|  |  | Prolapsed disc/slipped disc | 1312 |
|  |  | Spine arthritis/spondylitis | 1311 |
|  |  | Ankylosing spondylitis | 1313 |
|  |  | Back problem | 1294 |
|  |  | Osteoarthritis | 1465 |
|  |  | Gout | 1466 |
|  |  | Cervical spondylosis | 1478 |
|  |  | Trigeminal neuralgia | 1523 |
|  |  | Disc degeneration | 1533 |
|  |  | Trapped nerve/compressed nerve | 1257 |
| **24** | Osteoporosis | Osteoporosis | 1309 |
| **25** | Thyroid disorders | Thyroid problem (not cancer | 1224 |
|  |  | Hyperthroidism/thyrotoxicosis | 1225 |
|  |  | Hypothyroidism/myxoedema | 1226 |
|  |  | Grave’s disease | 1522 |
|  |  | Thyroid goitre | 1610 |
|  |  | Thyroititis | 1428 |
| **26** | Alcohol problems | Alcohol dependency | 1408 |
|  |  | Alcoholic liver disease/alcoholic cirrhosis | 1604 |
| **27** | Chronic kidney disease | Polycystic kidney | 1427 |
|  |  | Diabetic nephropathy | 1607 |
|  |  | Renal/kidney failure | 1192 |
|  |  | Renal failure requiring dialysis | 1193 |
|  |  | Renal failure not requiring dialysis | 1194 |
|  |  | Kidney nephropathy | 1519 |
|  |  | Immunoglobulin A (IgA) nephropathy | 1520 |
| **28** | Prostate disorders | Prostate problem (not cancer) | 1207 |
|  |  | Enlarged prostate | 1396 |
|  |  | Benign prostatic hypertrophy | 1516 |
| **29** | Glaucoma | Glaucoma | 1277 |
| **30** | Epilepsy | Epilepsy | 1264 |
| **31** | Dementia | Dementia/Alzheimer/cognitive impairment | 1263 |
| **32** | Psoriasis or eczema | Eczema/dermatitis | 1452 |
|  |  | Psoriasis | 1453 |
| **33** | Migraine | Migraine | 1265 |
| **34** | Chronic sinusitis | Chronic sinusitis | 1416 |
| **35** | Anorexia or bulimia | Anorexia, bulimia/other eating disorder | 1470 |
| **36** | Parkinson’s disease | Parkinson’s disease | 1262 |
| **37** | Multiple sclerosis | Multiple sclerosis | 1261 |
| **38** | Chronic fatigue syndrome | Chronic fatigue syndrome | 1482 |
| **39** | Endometriosis | Endometriosis | 1402 |
| **40** | Meniere disease | Meniere disease | 1421 |
| **41** | Pernicious anaemia | Pernicious anaemia | 1331 |
| **42** | Polycystic ovaries | Polycystic ovaries | 1350 |

*Self-report lifetime diagnosis by doctor recorded by nurse-led interview (UK Biobank data field ID: 20002), except cancer diagnosis which was reported by touch-screen questionnaire. The list of disease groupings was based on Barnett et al (2012).

**Table S4. Information of 142 SNPs for AF-PRS in UK Biobank**

| rsID | Position (hg19) | Prioritized genes | risk allele | reference allele | Effect size, β | Standard error |
| --- | --- | --- | --- | --- | --- | --- |
| rs10006327 | chr4:103890980 | SLC9B1 | C | T | 0.0357 | 0.0079 |
| rs10213171 | chr4:148937537 | ARHGAP10 | G | C | 0.0822 | 0.0155 |
| rs10458660 | chr10:77936576 | C10orf11 | G | A | 0.0551 | 0.0102 |
| rs10465885 | chr1:147232740 | GJA5 | C | T | 0.0262 | 0.0079 |
| rs10520260 | chr4:174447349 | HAND2, HAND2-AS1 | A | G | 0.0425 | 0.0086 |
| rs10741807 | chr11:20011445 | NAV2 | T | C | 0.0679 | 0.0093 |
| rs10749053 | chr10:112576695 | RBM20 | T | C | 0.0533 | 0.0116 |
| rs10753933 | chr1:203026214 | PPFIA4 | T | G | 0.0529 | 0.008 |
| rs10773657 | chr12:123327900 | HIP1R | C | A | 0.0649 | 0.012 |
| rs10804493 | chr3:111554426 | PHLDB2, PLCXD2 | A | G | 0.0507 | 0.0083 |
| rs10821415 | chr9:97713459 | C9orf3 | A | C | 0.0768 | 0.008 |
| rs10873298 | chr14:77426525 | IRF2BPL | C | T | 0.0295 | 0.0082 |
| rs11125871 | chr2:61470126 | USP34 | C | T | 0.0322 | 0.0081 |
| rs11156751 | chr14:32990437 | AKAP6 | C | T | 0.0677 | 0.0092 |
| rs11264280 | chr1:154862952 | KCNN3 | T | C | 0.1258 | 0.0085 |
| rs114904067 | chr4:112604821 | PITX2 | G | A | 0.0526 | 0.0268 |
| rs11590635 | chr1:49309764 | AGBL4 | A | G | 0.1731 | 0.0306 |
| rs11598047 | chr10:105342672 | NEURL1 | G | A | 0.1498 | 0.0104 |
| rs11614818 | chr12:56055815 | NACA | C | T | 0.0327 | 0.0082 |
| rs11658278 | chr17:38031164 | ZPBP2, GSDMB, ORMDL3 | T | C | 0.041 | 0.0079 |
| rs11773845 | chr7:116191301 | CAV1, CAV2 | A | C | 0.0924 | 0.008 |
| rs117984853 | chr6:149399100 | UST | T | G | 0.1064 | 0.0144 |
| rs118159104 | chr16:1676804 | RPL3L | G | T | 0.1784 | 0.0412 |
| rs12188351 | chr5:168386089 | SLIT3 | A | G | 0.0784 | 0.0169 |
| rs12245149 | chr10:65321147 | REEP3, NRBF2 | C | A | 0.0376 | 0.0079 |
| rs12426679 | chr12:76237987 | PHLDA1 | C | T | 0.0309 | 0.0079 |
| rs12604076 | chr17:76773638 | CYTH1, USP36 | T | C | 0.0369 | 0.0078 |
| rs12648245 | chr4:174641184 | HAND2, HAND2-AS1 | T | C | 0.0995 | 0.0152 |
| rs1278493 | chr3:135814009 | PPP2R3A | G | A | 0.0327 | 0.008 |
| rs12809354 | chr12:32978437 | PKP2 | C | T | 0.0553 | 0.0111 |
| rs12908004 | chr15:80676925 | ARNT2 | G | A | 0.0696 | 0.0106 |
| rs13195459 | chr6:122403559 | HSF2 | G | A | 0.0637 | 0.0083 |
| rs133902 | chr22:26164079 | MYO18B | T | C | 0.0478 | 0.0082 |
| rs138311480 | chr4:112454295 | PITX2 | C | T | 0.1196 | 0.0397 |
| rs140185678 | chr16:2003016 | RPL3L | A | G | 0.1439 | 0.0286 |
| rs1458038 | chr4:81164723 | FGF5 | T | C | 0.0406 | 0.0085 |
| rs146518726 | chr1:51535039 | MIR6500 | A | G | 0.1709 | 0.0235 |
| rs147301839 | chr15:57924714 | GCOM1/MYZAP | C | A | 0.3406 | 0.0617 |
| rs1532170 | chr4:112165212 | PITX2 | G | A | 0.0357 | 0.0084 |
| rs1545300 | chr1:112464004 | KCND3 | C | T | 0.05 | 0.0086 |
| rs1563304 | chr17:44874453 | WNT3 | T | C | 0.0669 | 0.0112 |
| rs17005647 | chr3:69406181 | FRMD4B | T | C | 0.0342 | 0.0082 |
| rs17380837 | chr12:26345526 | SSPN | C | T | 0.0439 | 0.0086 |
| rs1957021 | chr14:32924505 | AKAP6 | C | T | 0.0489 | 0.0094 |
| rs2012809 | chr5:128190363 | SLC27A6 | G | A | 0.05 | 0.0116 |
| rs2031522 | chr6:87821501 | CGA | A | G | 0.0441 | 0.0081 |
| rs2040862 | chr5:137419989 | WNT8A, NPY6R, MYOT, FAM13B | T | C | 0.1025 | 0.0103 |
| rs2274115 | chr9:139094773 | LHX3 | G | A | 0.0526 | 0.0091 |
| rs2288327 | chr2:179411665 | TTN, MIR548N, FKBP7, TTN-AS1 | G | A | 0.0825 | 0.0105 |
| rs2291437 | chr12:24715048 | LINC00477 | G | T | 0.0931 | 0.0121 |
| rs2359171 | chr16:73053022 | ZFHX3 | A | T | 0.1577 | 0.0101 |
| rs244017 | chr4:111255917 | PITX2 | T | G | 0.0189 | 0.0101 |
| rs2540949 | chr2:65284231 | CEP68 | A | T | 0.0625 | 0.008 |
| rs2738413 | chr14:64679960 | SYNE2, MIR548AZ, ESR2, MTHFD1 | A | G | 0.0704 | 0.0079 |
| rs2759301 | chr15:80994288 | ARNT2 | A | G | 0.042 | 0.0079 |
| rs2834618 | chr21:36119111 | LINC01426 | T | G | 0.101 | 0.0133 |
| rs28387148 | chr2:127433465 | GYPC | T | C | 0.0872 | 0.0136 |
| rs284277 | chr1:10790797 | CASZ1 | C | A | 0.0432 | 0.0082 |
| rs28439930 | chr5:173393111 | NKX2-5 | G | C | 0.0409 | 0.008 |
| rs2860482 | chr12:57105938 | NACA | A | C | 0.0476 | 0.0091 |
| rs2885697 | chr1:41544279 | SCMH1 | G | T | 0.0403 | 0.0082 |
| rs3176326 | chr6:36647289 | CDKN1A, PANDAR, PI16 | G | A | 0.068 | 0.0101 |
| rs337705 | chr5:113737062 | KCNN2 | G | T | 0.0583 | 0.0081 |
| rs34080181 | chr3:66454191 | LRIG1, SLC25A26 | G | A | 0.0371 | 0.0083 |
| rs34969716 | chr6:18210109 | KDM1B, DEK | A | G | 0.0651 | 0.0094 |
| rs35005436 | chr7:74134911 | GTF2I, LOC101926943, GTF2IRD2 | C | T | 0.0546 | 0.0119 |
| rs35176054 | chr10:105480387 | SH3PXD2A | A | T | 0.1281 | 0.0117 |
| rs35544454 | chr2:213266003 | ERBB4 | A | T | 0.0633 | 0.0103 |
| rs35569628 | chr13:113872712 | CUL4A | T | C | 0.0465 | 0.0095 |
| rs35620480 | chr8:11499908 | GATA4 | C | A | 0.051 | 0.011 |
| rs3820888 | chr2:201180023 | SPATS2L | C | T | 0.0599 | 0.008 |
| rs3853445 | chr4:111761487 | PITX2 | T | C | 0.1531 | 0.009 |
| rs3951016 | chr6:118559658 | SLC35F1, PLN | A | T | 0.0723 | 0.0079 |
| rs4073778 | chr1:116297758 | CASQ2 | A | C | 0.0493 | 0.008 |
| rs422068 | chr14:23864804 | MYH6, MYH7 | C | T | 0.0246 | 0.0084 |
| rs464901 | chr22:18597502 | TUBA8 | T | C | 0.0418 | 0.0086 |
| rs4871397 | chr8:124635197 | FBXO32 | G | C | 0.0829 | 0.0164 |
| rs4935786 | chr11:121661507 | SORL1 | T | A | 0.0362 | 0.0095 |
| rs4951258 | chr1:205691316 | NUCKS1, SLC41A1 | A | G | 0.0344 | 0.0079 |
| rs4963776 | chr12:24779491 | LINC00477 | G | T | 0.0808 | 0.0104 |
| rs4965430 | chr15:99268850 | IGF1R | C | G | 0.0397 | 0.0081 |
| rs4999127 | chr1:154714006 | KCNN3 | A | G | 0.08 | 0.0119 |
| rs55693294 | chr10:105277474 | NEURL1 | T | C | 0.0446 | 0.0177 |
| rs55734480 | chr7:14372009 | DGKB | A | G | 0.0419 | 0.0094 |
| rs55985730 | chr7:128417044 | OPN1SW, CALU | G | T | 0.0782 | 0.0177 |
| rs56181519 | chr2:175555714 | WIPF1 | C | T | 0.0576 | 0.0091 |
| rs56201652 | chr7:92278116 | CDK6 | G | A | 0.0563 | 0.0089 |
| rs577676 | chr1:170587340 | LINC01142 | C | T | 0.0977 | 0.0079 |
| rs60212594 | chr10:75414344 | SYNPO2L, NUDT13, MYOZ1, AGAP5 | G | C | 0.1272 | 0.0113 |
| rs60902112 | chr3:194800853 | XXYLT1 | T | C | 0.0388 | 0.0093 |
| rs61501369 | chr4:111524629 | PITX2 | T | C | 0.0788 | 0.0096 |
| rs62521286 | chr8:124551975 | FBXO32 | G | A | 0.1131 | 0.0161 |
| rs6462079 | chr7:28415827 | CREB5 | A | G | 0.0519 | 0.009 |
| rs6560886 | chr12:133150210 | FBRSL1 | C | T | 0.051 | 0.0109 |
| rs6580277 | chr5:142818123 | NR3C1 | G | A | 0.0759 | 0.0094 |
| rs6596717 | chr5:106427609 | LOC102467213 | C | A | 0.0356 | 0.008 |
| rs6689306 | chr1:154395946 | KCNN3 | A | G | 0.044 | 0.0081 |
| rs67249485 | chr4:111699685 | PITX2 | T | A | 0.3422 | 0.0094 |
| rs6747542 | chr2:70106832 | GMCL1, ANXA4 | T | C | 0.0602 | 0.0079 |
| rs6771054 | chr3:89489529 | EPHA3 | T | C | 0.0405 | 0.0081 |
| rs6790396 | chr3:38771925 | SCN10A, SCN5A | G | C | 0.0604 | 0.0081 |
| rs67969609 | chr2:145760353 | TEX41 | G | C | 0.0764 | 0.0147 |
| rs6829664 | chr4:114448656 | CAMK2D | G | A | 0.0638 | 0.009 |
| rs6850025 | chr4:111596360 | PITX2 | A | G | 0.1536 | 0.0182 |
| rs6891790 | chr5:172670745 | NKX2-5 | G | T | 0.0716 | 0.009 |
| rs6994744 | chr8:141740868 | PTK2 | C | A | 0.033 | 0.0079 |
| rs7096385 | chr10:69664881 | SIRT1, MYPN | T | C | 0.068 | 0.0152 |
| rs71454237 | chr12:70013415 | LRRC10 | G | A | 0.0613 | 0.01 |
| rs7170477 | chr15:64103777 | HERC1 | A | G | 0.0389 | 0.0085 |
| rs7225165 | chr17:1309850 | YWHAE, CRK, MYO1C | G | A | 0.0744 | 0.0135 |
| rs72700114 | chr1:170193825 | LINC01142 | C | G | 0.1698 | 0.0154 |
| rs72700118 | chr1:170194823 | METTL11B--KIFAP3 | A | C | 0.1162 | 0.012 |
| rs72811294 | chr17:12618680 | MYOCD | G | C | 0.0658 | 0.0126 |
| rs72926475 | chr2:86594487 | REEP1 | G | A | 0.0548 | 0.0121 |
| rs73041705 | chr3:24463235 | THRB | T | C | 0.0465 | 0.0088 |
| rs73241997 | chr14:35173775 | CFL2 | T | C | 0.0622 | 0.0109 |
| rs73366713 | chr6:16415751 | ATXN1 | G | A | 0.0923 | 0.012 |
| rs7373065 | chr3:38710315 | SCN10A, SCN5A | T | C | 0.1813 | 0.0299 |
| rs7374540 | chr3:38634142 | SCN10A, SCN5A | A | C | 0.027 | 0.008 |
| rs74022964 | chr15:73677264 | HCN4 | T | C | 0.1079 | 0.0106 |
| rs74884082 | chr14:73249419 | DPF3 | C | T | 0.0461 | 0.0093 |
| rs7508 | chr8:17913970 | ASAH1 | A | G | 0.0711 | 0.0088 |
| rs7529220 | chr1:22282619 | HSPG2 | C | T | 0.0686 | 0.0116 |
| rs7578393 | chr2:26165528 | KIF3C | T | C | 0.0615 | 0.0103 |
| rs76097649 | chr11:128764570 | KCNJ5 | A | G | 0.0988 | 0.015 |
| rs7612445 | chr3:179172979 | GNB4 | T | G | 0.0428 | 0.0099 |
| rs7650482 | chr3:12841804 | CAND2 | G | A | 0.0668 | 0.0082 |
| rs7687819 | chr4:113329345 | PITX2 | A | G | 0.02 | 0.0094 |
| rs77316573 | chr16:2265271 | RPL3L | T | C | 0.0383 | 0.0107 |
| rs775498 | chr12:70071513 | LRRC10 | G | A | 0.0362 | 0.0088 |
| rs7789146 | chr7:150661409 | KCNH2 | G | A | 0.0568 | 0.0103 |
| rs7834729 | chr8:21821778 | XPO7 | G | T | 0.0587 | 0.0123 |
| rs79187193 | chr1:147255831 | GJA5 | G | A | 0.1136 | 0.0179 |
| rs79399769 | chr4:111925656 | PITX2 | C | T | 0.1177 | 0.0296 |
| rs8088085 | chr18:48708548 | MEX3C | A | C | 0.0388 | 0.0079 |
| rs876727 | chr16:73067761 | ZFHX3 | T | G | 0.0745 | 0.0101 |
| rs883079 | chr12:114793240 | TBX5 | T | C | 0.0872 | 0.0087 |
| rs9401451 | chr6:122099152 | HSF2 | G | A | 0.0703 | 0.0129 |
| rs9506925 | chr13:23368943 | LINC00540, LINC00621, SGCG | T | C | 0.0365 | 0.009 |
| rs9899183 | chr17:7452977 | TNFSF12, TNFSF12-TNFSF13, SOX15, FXR2 | T | C | 0.0432 | 0.009 |
| rs9953366 | chr18:46474192 | SMAD7 | C | T | 0.0442 | 0.0087 |
| rs9963878 | chr18:48679522 | MEX3C | C | T | 0.0695 | 0.0142 |

**Table S5. Association between AF-PRS and Incident AF (n=440,365)**

|  | **Low PRS** | **Intermediate PRS** | **High PRS** | **Per SD increase** | ***P* trend** |
| --- | --- | --- | --- | --- | --- |
| **Incident rate per 10,000 py** | 33.3 | 48.3 | 77.0 |  |  |
| **Crude model** | 1 (ref.) | 1.45 (1.41, 1.50) | 2.33 (2.26, 2.40) | 1.48 (1.46, 1.49) | <0.0001 |
| **Adjusted model*** | 1 (ref.) | 1.48 (1.43, 1.52) | 2.43 (2.36, 2.50) | 1.51 (1.49, 1.53) | <0.0001 |

*Adjusted for age, sex, the first 10 primary components of ancestry, and genotype measurement batches.

**Table S6. Individual Components of Frailty Phenotype and Their Association with Incident Arrhythmia**

|  | **Model 1** | **Model 2** | **Model 3** |
| --- | --- | --- | --- |
| **AF** |  |  |  |
| Weight loss | 1.21 (1.17, 1.25) | 1.12 (1.09, 1.16) | 1.13 (1.09, 1.16) |
| Exhaustion | 1.44 (1.39, 1.49) | 1.15 (1.11, 1.19) | 1.11 (1.07, 1.14) |
| Low physical activity | 1.42 (1.37, 1.47) | 1.12 (1.07, 1.16) | 1.03 (0.996, 1.08) |
| Slow gait speed | 1.99 (1.93, 2.06) | 1.37 (1.33, 1.42) | 1.34 (1.30, 1.39) |
| Low grip strength | 1.25 (1.21, 1.28) | 1.04 (1.01, 1.07) | 1.00 (0.97, 1.03) |
| **Bradyarrhythmias** |  |  |  |
| Weight loss | 1.27 (1.21, 1.33) | 1.17 (1.11, 1.22) | 1.17 (1.12, 1.23) |
| Exhaustion | 1.47 (1.39, 1.55) | 1.18 (1.11, 1.24) | 1.13 (1.07, 1.20) |
| Low physical activity | 1.34 (1.26, 1.42) | 1.07 (1.005, 1.13) | 0.99 (0.93, 1.05) |
| Slow gait speed | 1.90 (1.80, 1.99) | 1.33 (1.26, 1.41) | 1.30 (1.22, 1.37) |
| Low grip strength | 1.34 (1.28, 1.40) | 1.13 (1.08, 1.18) | 1.10 (1.05, 1.15) |
| **Conduction system diseases** |  |  |  |
| Weight loss | 1.24 (1.19, 1.30) | 1.12 (1.07, 1.17) | 1.13 (1.08, 1.18) |
| Exhaustion | 1.53 (1.46, 1.61) | 1.17 (1.11, 1.23) | 1.11 (1.05, 1.17) |
| Low physical activity | 1.56 (1.48, 1.65) | 1.20 (1.14, 1.27) | 1.12 (1.05, 1.18) |
| Slow gait speed | 2.06 (1.96, 2.16) | 1.36 (1.29, 1.43) | 1.29 (1.22, 1.36) |
| Low grip strength | 1.40 (1.34, 1.46) | 1.16 (1.11, 1.21) | 1.12 (1.07, 1.17) |
| **Supraventricular arrhythmias** |  |  |  |
| Weight loss | 1.08 (0.99, 1.17) | 1.01 (0.93, 1.10) | 1.01 (0.93, 1.10) |
| Exhaustion | 1.42 (1.30, 1.55) | 1.22 (1.11, 1.33) | 1.18 (1.08, 1.30) |
| Low physical activity | 1.13 (1.02, 1.26) | 0.99 (0.89, 1.10) | 0.91 (0.82, 1.02) |
| Slow gait speed | 1.66 (1.51, 1.82) | 1.33 (1.20, 1.48) | 1.32 (1.19, 1.47) |
| Low grip strength | 1.18 (1.09, 1.28) | 1.06 (0.97, 1.15) | 1.03 (0.94, 1.12) |
| **Ventricular arrhythmias** |  |  |  |
| Weight loss | 1.16 (1.07, 1.26) | 1.05 (0.97, 1.14) | 1.06 (0.97, 1.15) |
| Exhaustion | 1.46 (1.34, 1.60) | 1.12 (1.02, 1.22) | 1.05 (0.95, 1.15) |
| Low physical activity | 1.62 (1.48, 1.78) | 1.25 (1.14, 1.38) | 1.15 (1.04, 1.26) |
| Slow gait speed | 2.14 (1.97, 2.32) | 1.46 (1.33, 1.60) | 1.37 (1.25, 1.51) |
| Low grip strength | 1.47 (1.36, 1.58) | 1.23 (1.14, 1.33) | 1.19 (1.10, 1.28) |

Model 1, included age, sex, race, Townsend deprivation index, and assessment centers.

Model 2, included Model 1 plus alcohol consumption, smoking status, sedentary behavior, BMI, the number of long-term morbidities, AF-PRS, the first 10 primary components of ancestry, and genotype measurement batches.

Model 3, included Model 2 plus five frailty components (mutual adjustment).

**Table S7. Risk of Incident AF According to Frailty Status within the AF-PRS Category (n=440,365)**

|  | Low PRS | Intermediate PRS | High PRS | *P* for interaction* |
| --- | --- | --- | --- | --- |
| Non-Frailty | 1.00 (ref.) | 1.00 (ref.) | 1.00 (ref.) | <0.0001 |
| Pre-frailty | 1.16 (1.10, 1.23) | 1.14 (1.09, 1.19) | 1.09 (1.05, 1.13) |  |
| Frailty | 1.53 (1.37, 1.71) | 1.40 (1.27, 1.54) | 1.36 (1.26, 1.48) |  |

Model adjusted for age, sex, Townsend deprivation index, assessment centers, alcohol consumption, smoking status, sedentary behavior, BMI, the number of long-term morbidities, genotyping array, and the first 10 principal components of ancestry.

**P* for the interaction between frailty status and AF-PRS categories.

**Table S8. Association between Frailty and Incident AF by Subgroups**

| **Incident AF** | **N** | **Non-frailty** | **Pre-frailty** | **Frailty** | ***P* for interaction** |
| --- | --- | --- | --- | --- | --- |
| **Age** |  |  |  |  |  |
| <55 years | 181,939 | 1.00 (ref.) | 1.18 (1.10, 1.27) | 1.58 (1.36, 1.84) | 0.019 |
| 55 to 64 years | 196,116 | 1.00 (ref.) | 1.11 (1.07, 1.15) | 1.38 (1.28, 1.49) |  |
| ≥65 years | 86,099 | 1.00 (ref.) | 1.12 (1.08, 1.17) | 1.46 (1.35, 1.59) |  |
| **Sex** |  |  |  |  |  |
| Female | 253,752 | 1.00 (ref.) | 1.18 (1.14, 1.23) | 1.49 (1.38, 1.61) | 0.0069 |
| Male | 210,402 | 1.00 (ref.) | 1.08 (1.05, 1.12) | 1.42 (1.32, 1.52) |  |
| **Townsend deprivation index** |  |  |  |  |  |
| Below median value (low deprivation) | 232,064 | 1.00 (ref.) | 1.09 (1.05, 1.13) | 1.35 (1.23, 1.49) | 0.0004 |
| Above median value (high deprivation) | 232,090 | 1.00 (ref.) | 1.16 (1.12, 1.20) | 1.50 (1.41, 1.60) |  |
| **BMI category** |  |  |  |  |  |
| <25.0 kg/m^2^ | 155,115 | 1.00 (ref.) | 1.16 (1.11, 1.22) | 1.61 (1.40, 1.85) | 0.53 |
| 25.0 to 29.9 kg/m^2^ | 197,802 | 1.00 (ref.) | 1.11 (1.07, 1.16) | 1.47 (1.34, 1.62) |  |
| ≥30.0 kg/m^2^ | 111,237 | 1.00 (ref.) | 1.15 (1.10, 1.20) | 1.54 (1.43, 1.65) |  |
| **The number of long-term morbidities** |  |  |  |  |  |
| None | 160,004 | 1.00 (ref.) | 1.07 (1.01, 1.13) | 1.47 (1.15, 1.87) | <0.0001 |
| One | 154,262 | 1.00 (ref.) | 1.13 (1.08, 1.18) | 1.45 (1.26, 1.65) |  |
| Two or more | 149,888 | 1.00 (ref.) | 1.17 (1.13, 1.21) | 1.57 (1.48, 1.66) |  |

Values are presented as HR (95% CI) and adjusted for age, sex, race, Townsend deprivation index, assessment centers, alcohol consumption, smoking status, sedentary behavior, BMI, the number of long-term morbidities, genotyping array, and the first 10 principal components of ancestry.

**Table S9. Association between Frailty and Incident Bradyarrhythmia by Subgroups**

| **Incident bradyarrhythmia** | **N** | **Non-frailty** | **Pre-frailty** | **Frailty** | ***P* for interaction** |
| --- | --- | --- | --- | --- | --- |
| **Age** |  |  |  |  |  |
| <55 years | 181,939 | 1.00 (ref.) | 1.21 (1.10, 1.35) | 1.39 (1.10, 1.76) | 0.0014 |
| 55 to 64 years | 196,116 | 1.00 (ref.) | 1.15 (1.09, 1.22) | 1.59 (1.42, 1.79) |  |
| ≥65 years | 86,099 | 1.00 (ref.) | 1.15 (1.09, 1.22) | 1.43 (1.25, 1.63) |  |
| **Sex** |  |  |  |  |  |
| Female | 253,752 | 1.00 (ref.) | 1.18 (1.10, 1.26) | 1.61 (1.41, 1.82) | 0.16 |
| Male | 210,402 | 1.00 (ref.) | 1.15 (1.10, 1.21) | 1.46 (1.32, 1.62) |  |
| **Townsend deprivation index** |  |  |  |  |  |
| Below median value (low deprivation) | 232,064 | 1.00 (ref.) | 1.19 (1.13, 1.26) | 1.42 (1.23, 1.65) | 0.74 |
| Above median value (high deprivation) | 232,090 | 1.00 (ref.) | 1.14 (1.08, 1.20) | 1.56 (1.41, 1.72) |  |
| **BMI category** |  |  |  |  |  |
| <25.0 kg/m^2^ | 155,115 | 1.00 (ref.) | 1.17 (1.08, 1.27) | 1.96 (1.58, 2.43) | 0.86 |
| 25.0 to 29.9 kg/m^2^ | 197,802 | 1.00 (ref.) | 1.17 (1.11, 1.24) | 1.44 (1.24, 1.67) |  |
| ≥30.0 kg/m^2^ | 111,237 | 1.00 (ref.) | 1.17 (1.09, 1.25) | 1.57 (1.40, 1.75) |  |
| **The number of long-term morbidities** |  |  |  |  |  |
| None | 160,004 | 1.00 (ref.) | 1.24 (1.14, 1.36) | 1.52 (1.03, 2.25) | 0.57 |
| One | 154,262 | 1.00 (ref.) | 1.14 (1.07, 1.22) | 1.52 (1.24, 1.87) |  |
| Two or more | 149,888 | 1.00 (ref.) | 1.18 (1.11, 1.24) | 1.64 (1.50, 1.80) |  |

Values are presented as HR (95% CI) and adjusted for age, sex, race, Townsend deprivation index, assessment centers, alcohol consumption, smoking status, sedentary behavior, BMI, the number of long-term morbidities, genotyping array, and the first 10 principal components of ancestry.

**Table S10. Association between Frailty and Incident Conduction System Diseases by Subgroups**

| **Incident conduction system diseases** | **N** | **Non-frailty** | **Pre-frailty** | **Frailty** | ***P* for interaction** |
| --- | --- | --- | --- | --- | --- |
| **Age** |  |  |  |  |  |
| <55 years | 181,939 | 1.00 (ref.) | 1.15 (1.04, 1.27) | 1.52 (1.23, 1.88) | 0.24 |
| 55 to 64 years | 196,116 | 1.00 (ref.) | 1.22 (1.15, 1.29) | 1.56 (1.40, 1.75) |  |
| ≥65 years | 86,099 | 1.00 (ref.) | 1.21 (1.14, 1.28) | 1.56 (1.38, 1.76) |  |
| **Sex** |  |  |  |  |  |
| Female | 253,752 | 1.00 (ref.) | 1.20 (1.13, 1.28) | 1.54 (1.37, 1.74) | 0.10 |
| Male | 210,402 | 1.00 (ref.) | 1.20 (1.14, 1.26) | 1.60 (1.45, 1.76) |  |
| **Townsend deprivation index** |  |  |  |  |  |
| Below median value (low deprivation) | 232,064 | 1.00 (ref.) | 1.23 (1.17, 1.30) | 1.52 (1.33, 1.75) | 0.39 |
| Above median value (high deprivation) | 232,090 | 1.00 (ref.) | 1.18 (1.12, 1.24) | 1.59 (1.45, 1.75) |  |
| **BMI category** |  |  |  |  |  |
| <25.0 kg/m^2^ | 155,115 | 1.00 (ref.) | 1.19 (1.09, 1.29) | 1.93 (1.58, 2.35) | 0.14 |
| 25.0 to 29.9 kg/m^2^ | 197,802 | 1.00 (ref.) | 1.21 (1.14, 1.28) | 1.64 (1.43, 1.87) |  |
| ≥30.0 kg/m^2^ | 111,237 | 1.00 (ref.) | 1.22 (1.14, 1.31) | 1.57 (1.41, 1.75) |  |
| **The number of long-term morbidities** |  |  |  |  |  |
| None | 160,004 | 1.00 (ref.) | 1.26 (1.15, 1.38) | 1.56 (1.06, 2.27) | 0.56 |
| One | 154,262 | 1.00 (ref.) | 1.16 (1.08, 1.24) | 1.74 (1.43, 2.10) |  |
| Two or more | 149,888 | 1.00 (ref.) | 1.25 (1.19, 1.32) | 1.76 (1.61, 1.91) |  |

Values are presented as HR (95% CI) and adjusted for age, sex, race, Townsend deprivation index, assessment centers, alcohol consumption, smoking status, sedentary behavior, BMI, the number of long-term morbidities, genotyping array, and the first 10 principal components of ancestry.

**Table S11. Association between Frailty and Incident Supraventricular Arrhythmias by Subgroups**

| **Incident supraventricular arrhythmias** | **N** | **Non-frailty** | **Pre-frailty** | **Frailty** | ***P* for interaction** |
| --- | --- | --- | --- | --- | --- |
| **Age** |  |  |  |  |  |
| <55 years | 181,939 | 1.00 (ref.) | 1.16 (1.02, 1.32) | 1.14 (0.81, 1.61) | 0.47 |
| 55 to 64 years | 196,116 | 1.00 (ref.) | 1.11 (1.01, 1.22) | 1.28 (1.04, 1.59) |  |
| ≥65 years | 86,099 | 1.00 (ref.) | 1.10 (0.97, 1.25) | 1.35 (1.03, 1.77) |  |
| **Sex** |  |  |  |  |  |
| Female | 253,752 | 1.00 (ref.) | 1.16 (1.06, 1.27) | 1.40 (1.16, 1.70) | 0.17 |
| Male | 210,402 | 1.00 (ref.) | 1.08 (0.98, 1.19) | 1.14 (0.89, 1.46) |  |
| **Townsend deprivation index** |  |  |  |  |  |
| Below median value (low deprivation) | 232,064 | 1.00 (ref.) | 1.15 (1.04, 1.26) | 1.10 (0.82, 1.47) | 0.47 |
| Above median value (high deprivation) | 232,090 | 1.00 (ref.) | 1.10 (1.01, 1.21) | 1.35 (1.13, 1.62) |  |
| **BMI category** |  |  |  |  |  |
| <25.0 kg/m^2^ | 155,115 | 1.00 (ref.) | 1.16 (1.03, 1.31) | 2.09 (1.54, 2.82) | 0.024 |
| 25.0 to 29.9 kg/m^2^ | 197,802 | 1.00 (ref.) | 1.07 (0.97, 1.18) | 1.26 (0.97, 1.64) |  |
| ≥30.0 kg/m^2^ | 111,237 | 1.00 (ref.) | 1.17 (1.03, 1.33) | 1.11 (0.88, 1.40) |  |
| **The number of long-term morbidities** |  |  |  |  |  |
| None | 160,004 | 1.00 (ref.) | 0.98 (0.85, 1.12) | 0.60 (0.25, 1.45) | 0.053 |
| One | 154,262 | 1.00 (ref.) | 1.24 (1.11, 1.39) | 1.77 (1.27, 2.46) |  |
| Two or more | 149,888 | 1.00 (ref.) | 1.15 (1.04, 1.27) | 1.41 (1.19, 1.68) |  |

Values are presented as HR (95% CI) and adjusted for age, sex, race, Townsend deprivation index, assessment centers, alcohol consumption, smoking status, sedentary behavior, BMI, the number of long-term morbidities, genotyping array, and the first 10 principal components of ancestry.

**Table S12. Association between Frailty and Incident Ventricular Arrhythmia by Subgroups**

| **Incident ventricular arrhythmias** | **N** | **Non-frailty** | **Pre-frailty** | **Frailty** | ***P* for interaction** |
| --- | --- | --- | --- | --- | --- |
| **Age** |  |  |  |  |  |
| <55 years | 181,939 | 1.00 (ref.) | 1.17 (1.01, 1.35) | 1.43 (1.05, 1.96) | 0.81 |
| 55 to 64 years | 196,116 | 1.00 (ref.) | 1.17 (1.06, 1.29) | 1.54 (1.28, 1.86) |  |
| ≥65 years | 86,099 | 1.00 (ref.) | 1.28 (1.15, 1.43) | 1.80 (1.44, 2.25) |  |
| **Sex** |  |  |  |  |  |
| Female | 253,752 | 1.00 (ref.) | 1.23 (1.09, 1.39) | 1.68 (1.36, 2.09) | 0.89 |
| Male | 210,402 | 1.00 (ref.) | 1.19 (1.10, 1.29) | 1.59 (1.35, 1.87) |  |
| **Townsend deprivation index** |  |  |  |  |  |
| Below median value (low deprivation) | 232,064 | 1.00 (ref.) | 1.22 (1.11, 1.35) | 1.93 (1.53, 2.44) | 0.26 |
| Above median value (high deprivation) | 232,090 | 1.00 (ref.) | 1.19 (1.09, 1.31) | 1.52 (1.29, 1.78) |  |
| **BMI category** |  |  |  |  |  |
| <25.0 kg/m^2^ | 155,115 | 1.00 (ref.) | 1.28 (1.12, 1.46) | 1.78 (1.29, 2.44) | 0.096 |
| 25.0 to 29.9 kg/m^2^ | 197,802 | 1.00 (ref.) | 1.22 (1.10, 1.34) | 1.75 (1.40, 2.18) |  |
| ≥30.0 kg/m^2^ | 111,237 | 1.00 (ref.) | 1.15 (1.02, 1.29) | 1.50 (1.24, 1.81) |  |
| **The number of long-term morbidities** |  |  |  |  |  |
| None | 160,004 | 1.00 (ref.) | 1.17 (1.01, 1.36) | 1.40 (0.74, 2.63) | 0.040 |
| One | 154,262 | 1.00 (ref.) | 1.17 (1.04, 1.31) | 1.69 (1.22, 2.33) |  |
| Two or more | 149,888 | 1.00 (ref.) | 1.30 (1.18, 1.43) | 1.87 (1.62, 2.18) |  |

Values are presented as HR (95% CI) and adjusted for age, sex, race, Townsend deprivation index, assessment centers, alcohol consumption, smoking status, sedentary behavior, BMI, the number of long-term morbidities, genotyping array, and the first 10 principal components of ancestry.

**Table S13.** **Association between Frailty and Incident Arrhythmias Excluding Those Who Developed** **Arrhythmias or Died within Two Years of Follow-up** **(n=458,781)**

|  | **Non-frailty** | **Pre-frailty** | **Frailty** |
| --- | --- | --- | --- |
| AF | 1 (ref.) | 1.11 (1.08, 1.14) | 1.40 (1.33, 1.48) |
| Bradyarrhythmias | 1 (ref.) | 1.15 (1.11, 1.20) | 1.48 (1.35, 1.61) |
| Conduction system diseases | 1 (ref.) | 1.19 (1.15, 1.24) | 1.53 (1.41, 1.65) |
| Supraventricular arrhythmias | 1 (ref.) | 1.10 (1.02, 1.18) | 1.25 (1.06, 1.46) |
| Ventricular arrhythmias | 1 (ref.) | 1.18 (1.10, 1.27) | 1.52 (1.32, 1.75) |

Values are presented as HR (95% CI) and adjusted for age, sex, race, Townsend deprivation index, assessment centers, alcohol consumption, smoking status, sedentary behavior, BMI, the number of long-term morbidities, AF-PRS, the first 10 primary components of ancestry, and genotype measurement batches.

**Table S14.** **Association between Frailty and Incident Arrhythmias Using Fine** **& Gray Models for Competing Risk**

|  | **Non-frailty** | **Pre-frailty** | **Frailty** |
| --- | --- | --- | --- |
| AF | 1 (ref.) | 1.10 (1.07, 1.12) | 1.33 (1.27, 1.41) |
| Bradyarrhythmias | 1 (ref.) | 1.13 (1.09, 1.17) | 1.36 (1.26, 1.48) |
| Conduction system diseases | 1 (ref.) | 1.17 (1.13, 1.22) | 1.42 (1.31, 1.53) |
| Supraventricular arrhythmias | 1 (ref.) | 1.11 (1.04, 1.18) | 1.21 (1.04, 1.40) |
| Ventricular arrhythmias | 1 (ref.) | 1.18 (1.10, 1.26) | 1.47 (1.29, 1.68) |

Values are presented as HR (95% CI) and adjusted for age, sex, race, Townsend deprivation index, assessment centers, alcohol consumption, smoking status, sedentary behavior, BMI, the number of long-term morbidities, AF-PRS, the first 10 primary components of ancestry, and genotype measurement batches.

**Figure S1. Flow chart**

**
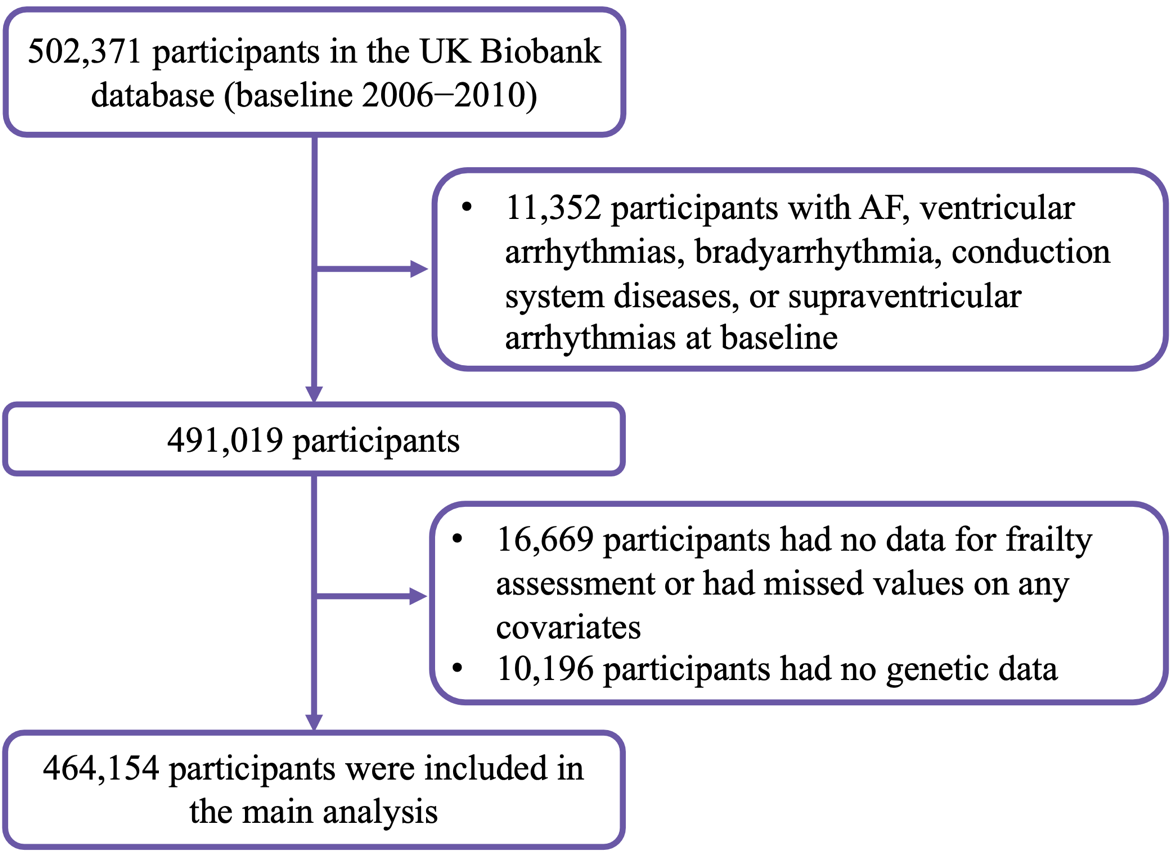
**

**Figure S2. The Curve of Density Distribution of AF-PRS**


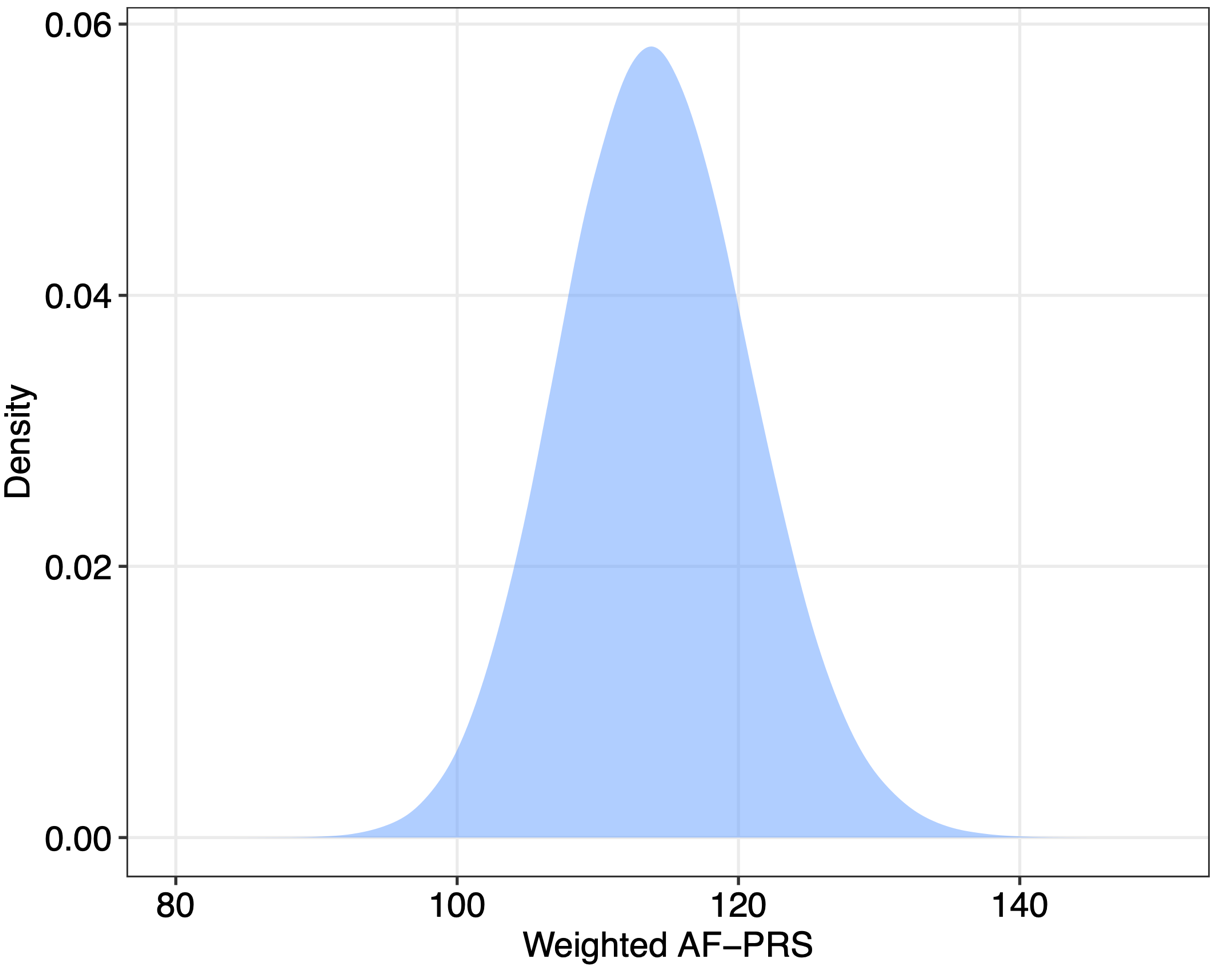


Supplementary references

S1. Jiang R, Noble S, Sui J, Yoo K, Rosenblatt M, Horien C, et al. Associations of physical frailty with health outcomes and brain structure in 483 033 middle-aged and older adults: a population-based study from the UK Biobank. Lancet Digit Health 2023.

S2. Barnett K, Mercer SW, Norbury M, Watt G, Wyke S, Guthrie B. Epidemiology of multimorbidity and implications for health care, research, and medical education: a cross-sectional study. Lancet 2012;380:37-43.

S3. Tajik B, Voutilainen A, Lyytinen A, Kauhanen J, Lip GYH, Tuomainen TP, Isanejad M. Frailty Predicts Incident Atrial Fibrillation in Women but Not in Men: The Kuopio Ischaemic Heart Disease Risk Factor Study. Cardiology. 2023;148(6):574-580.

S4. Orkaby AR, Kornej J, Lubitz SA, McManus DD, Travison TG, Sherer JA, Trinquart L, Murabito JM, Benjamin EJ, Preis SR. Association Between Frailty and Atrial Fibrillation in Older Adults: The Framingham Heart Study Offspring Cohort. J Am Heart Assoc. 2021 Jan 5;10(1):e018557.

S5. Hoogendijk EO, Afilalo J, Ensrud KE, Kowal P, Onder G, Fried LP. Frailty: implications for clinical practice and public health. Lancet 2019;394:1365-1375.

S6. Yang MT, Wu YW, Chan DC, Chien MY. The relationship between atrial fibrillation and frailty in community-dwelling older adults. Arch Gerontol Geriatr 2020;90:104103.

S7. Bhaskaran K, Dos-Santos-Silva I, Leon DA, Douglas IJ, Smeeth L. Association of BMI with overall and cause-specific mortality: a population-based cohort study of 3.6 million adults in the UK. Lancet Diabetes Endocrinol 2018;6:944-953.

S8. American Diabetes Association Professional Practice Committee. 13. Older Adults: Standards of Medical Care in Diabetes-2022. Diabetes Care. 2022 Jan 1;45(Suppl 1):S195-S207.

S9. Assar ME, Laosa O, Rodriguez ML. Diabetes and frailty. Curr Opin Clin Nutr Metab Care 2019;22:52-57.

S10. Minagawa Y, Saito Y. Subjective Well-Being and Active Life Expectancy in Japan: Evidence From a Longitudinal Study. Innov Aging 2023;7:igac075.

S11. Meinow B, Li P, Jasilionis D, Oksuzyan A, Sundberg L, Kelfve S, et al. Trends over two decades in life expectancy with complex health problems among older Swedes: implications for the provision of integrated health care and social care. BMC Public Health 2022;22:759.

S12. Khurshid S, Choi SH, Weng LC, Wang EY, Trinquart L, Benjamin EJ, et al. Frequency of Cardiac Rhythm Abnormalities in a Half Million Adults. Circ Arrhythm Electrophysiol 2018;11:e006273.
